# Queries on Cancer

**Published:** 1811-02

**Authors:** 


### *To the Editors of the Medical and Physical Journal.*

### *Queries on Cancer.*

GENTLEMEN,

PERMIT me, by means of your Journal, to request from such of your Readers as have paid attention to the Diseases of Animals, Replies to some or all of the following Queries, which, I doubt not, will be acceptable to others of your Readers, as well as to

Your's, &c.

S. M.

Is Cancer a disease to which animals are liable ?

What animals are most liable to Cancer ?

What parts of the animal are mostly attacked by Cancer ?

What are the diagnostics of Cancer in animals ?

Is the Cancer of animals characterized by the *firm white lines* of *Dr. Baillie;* the *firm whitish bands* of *Mr. Abernethy;* the *ligamentous bands of a whitish colour* of *Mr. Home ?*

Do any medicines relieve the Cancer of animals ?

Is the Cancer of animals curable ; and by what means ?

Does Cancer ever attack the uterus of animals ?

The uterus of women is liable to *very different* diseases, which pass under the common name of Cancer : Is the uterus of animals equally liable to diseases of these different kinds ?

